# Leukocyte cell population data in patients with cardiac surgery and cardiopulmonary bypass: A potential readily available tool to monitor immunity

**DOI:** 10.3389/fimmu.2022.1101937

**Published:** 2023-01-19

**Authors:** Maxime Nguyen, Laure Stiel, Adrien Guilloteau, Pierre-Alain Bahr, David Masson, Charles Thomas, Mathieu Blot, Julien Guy, Cécile Fontaine, Bastien Durand, Belaid Bouhemad, Pierre-Grégoire Guinot

**Affiliations:** ^1^ Department of Anaesthesiology and Intensive Care, Dijon University Hospital, Dijon, France; ^2^ University of Burgundy and Franche-Comté, LNC UMR1231, Dijon, France; ^3^ INSERM, LNC UMR1231, Dijon, France; ^4^ LipSTIC LabEx, Dijon, France; ^5^ Service de Réanimation Médicale, Groupe Hospitalier de la Région Mulhouse Sud Alsace, Mulhouse, France; ^6^ Clinical Research Support Unit, Dijon University Hospital, Dijon, France; ^7^ Department of Infectiology, Dijon University Hospital, Dijon, France; ^8^ Cytometry Core Facility, University of Burgundy Franche-Comté, Dijon, France

**Keywords:** biomarker, cell population data, cardiac surgery, cardiopulmonary bypass, inflammation, post-operative outcome, immunity, acute kidney injury

## Abstract

**Purpose:**

Cardiac surgery with cardiopulmonary bypass triggers sterile inflammation that is responsible for post-operative morbidity. Automated flow cytometry devices used for leucocyte count provide cell population data (CPD) regarding fluorescence intensity, size and granularity of leukocytes that have never been studied in the context of sterile inflammation. Our objective was to explore leukocyte cell population data in patients undergoing cardiac surgery with cardiopulmonary bypass in order to determine whether CPD could be used to monitor immune cell activation.

**Methods:**

This is an ancillary study of a cohort of patients undergoing cardiac surgery with cardiopulmonary bypass. Cell population data (CPD) extracted from a routine automated flow cytometer were analyzed (Fluorescence targeted to nucleic acids). The time points of interest were: pre-operative, postoperative and 5 days after surgery. The variations in those parameters were studied. Data were then compared between patients according to the occurrence of a composite criteria (supra-ventricular arrythmia, stroke, acute renal failure, and/or death).

**Results:**

Data from 1453 patients were analyzed. The neutrophil count, fluorescence granularity (NE-SCC), intensity (NE-SFL) and size (NE-FSC) increased with surgery. Heterogeneity of neutrophils decreased in terms of fluorescence granularity (NE-WX) and size (NE-WZ) but increased in terms of intensity (NE-WY). The lymphocyte count decreased with surgery. While fluorescence granularity (LY-X) and size increased (LY-Z), Lymphocyte intensity decreased (LY-Y). Lymphocytes were less heterogeneous in terms of their granularity, size and intensity after surgery (LY-WX, LY-WY, LY-WZ). Patients who developed the composite complication criteria had a higher pre-operative neutrophil count (5.08 [3.89;6.95] vs 4.76 [3.60;6.13], p = 0.02; AUC = 0.56 [0.51;0.60]), and more heterogeneous neutrophils in terms of fluorescence granularity (NE-WX, AUC = 0.57 [0.52;0.62]) and intensity (NE-WY, AUC 0.61 [0.56;0.65]). Those patients also had lower pre-operative lymphocyte count (1.49 [1.10;1.14] vs 1.81 [1.39;2.39], p<0.01, AUC = 0.61 [0.57;0.66]) and fluorescence granularity (LY-X, AUC = 0.57 [0.53;0.62]). NE-WX, NE-WY and LY-X were associated with post-operative complications after adjustment on the EuroSCORE 2 (adjusted odd ratio of 1.01 [1.00;1.02]; 1.01 [1.00;1.01] and 1.08 [1.02;1.15] respectively).

**Conclusion:**

Cardiac surgery with cardiopulmonary bypass was associated with substantial alterations of CPD probably reflecting leukocytes activation in sterile inflammation. Pre-operative NE-WX, NE-WY and LY-X biomarkers levels were associated with post-operative complications, independently of the EuroSCORE 2. Such routine, unexploited and low cost parameters might represent useful tools likely to monitor immune function and predict outcomes for patients undergoing cardiac surgery. Our findings requires validation on a larger external cohort.

## Introduction

Immune dysregulation is a major issue in critically ill patients and biomarkers for the monitoring of the immune function are urgently needed in order to implement therapies targeting immunity (1). Cardiac surgery with cardiopulmonary bypass (CPB) is performed daily for the treatment of cardiovascular conditions. Although the procedure of CPB has been improved, this surgery is associated with high incidences of complications and mortality. Cardiac surgery with CPB represents a major aggression (to the human body) and the sterile inflammatory response triggered by CPB drives post-operative complications (2).

In addition to cell count, automated hematology analyzers measure cell population data (CPD) that do not appear on traditional reports, meaning this information remains unused. These parameters are directly linked to leukocyte characteristics (granularity, size and fluorescence) and in patients with sepsis (3) it has been suggested that these CPD reflect leukocyte activation (4). However, they have never been studied in the context of sterile inflammation such as cardiac surgery with CPB.

The objective of the present study was to explore CPD as a potential marker of immune function in sterile inflammation by following the changing patterns of leukocyte CPD in patients undergoing cardiac surgery and cardiopulmonary bypass and by reporting associations between leukocyte CPD and post-operative complications.

We hypothesized that CPD would be highly modified by cardiac surgery and cardiopulmonary bypass (reflecting the activation of leukocytes) and that pre-operative levels of those parameters reflecting a pre-operative pro-inflammatory state would be associated with post-operative complications.

## Material and methods

### Patients

This is an ancillary analysis of a prospective cohort that aimed to study risk factor for vasoplegia in patients undergoing cardiac surgery with cardiopulmonary bypass. Patients were recruited between October 2017 and July 2021.The inclusion criteria were as follows: all adult patients undergoing cardiac surgery with cardiopulmonary bypass, covered by a social insurance system, who have given oral consent to participate after receiving full information and with at least one blood count at any of the time points of interest. Exclusion criteria were: cardiac graft, pre-operative extracorporeal life support or membrane oxygenation, mechanical cardiac assistance and patient deprived of liberty. Only patients included in Dijon university hospital were considered for the present analysis.

This report was drafted in accordance with the STROBE statement (5). This study was approved by the Institutional Review Board (IRB 00010254 - 2022 - 034). All patients received written information of their inclusion in the study.

### Protocol

Cytometry data were retrospectively extracted from the automated hematology analyzer (Sysmex XN-200, Sysmex, France). No blood sampling was mandatory for the study as blood cell count is usually monitored daily after cardiac surgery in our centre. Those data were merged with the data from the prospective cohort (ClinicalTrials.gov Identifier: NCT03281317). Three time points of interest were defined: pre-operative, post-operative (on the day of surgery) and 5 days after surgery. We choose the pre-operative and post-operative time points to capture the modifications of CPD induce by the surgery. Day 5 was chosen because it allowed to describe the evolution of CPD with the resolution of inflammation without too many lost too follow-up patients.

**Figure 1:**
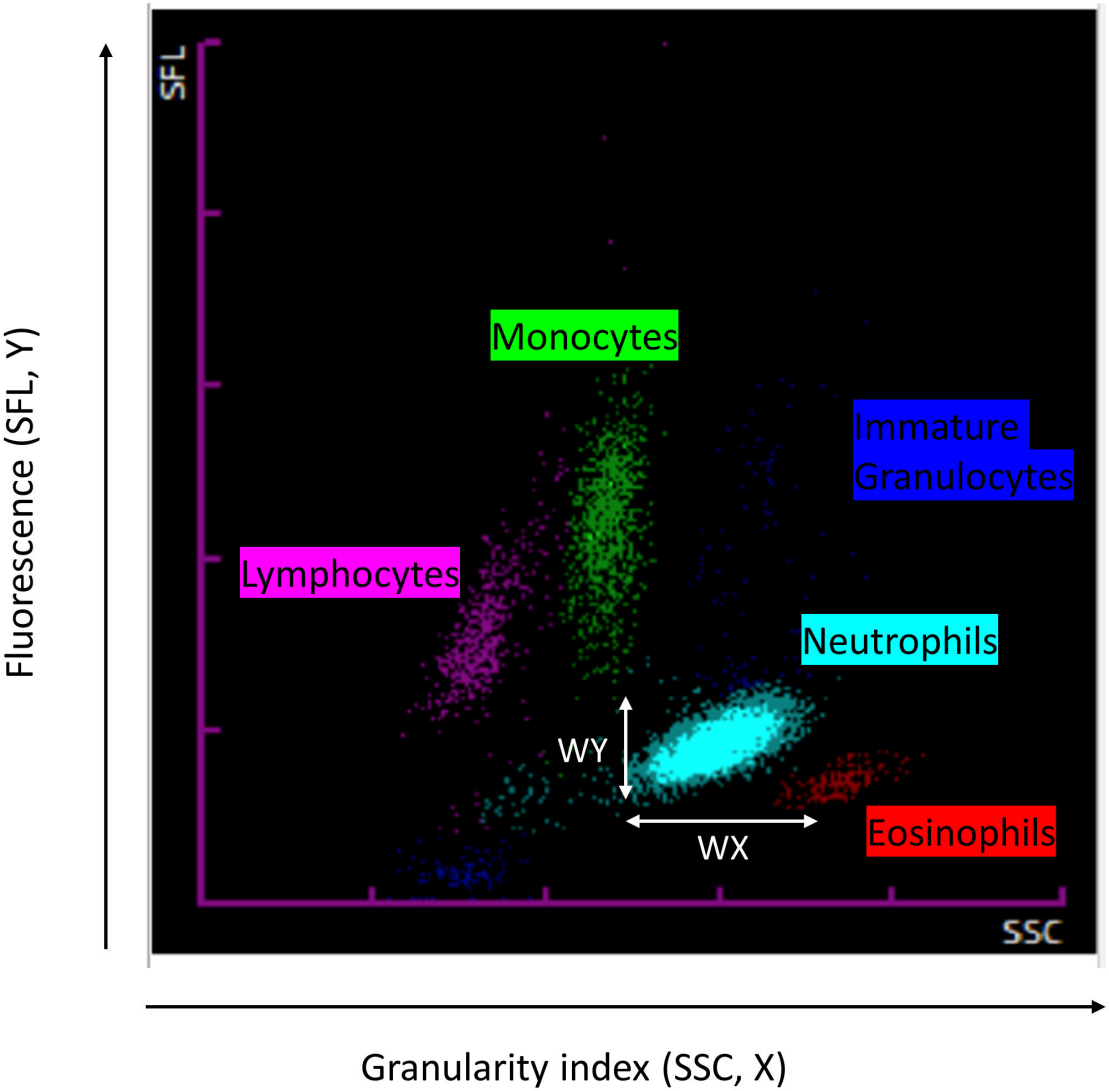
Cell population data parameters as visualized on the analyzer SCC: Granularity, SFL: Fluorescence.

Blood cell count (CBC) was measured on an automated Sysmex XN2000 analyzer equipped with impedance and fluorescence flow cytometry devices. The optical system of the XN2000 employs a red diode laser producing a light beam. Cell permeabilization is performed by treatment with specific lysis reagents allowing specific dyes to enter the cells, where they bind to nucleic acids in the cytoplasmic organelles and the nucleus. Three mains signals are recorded (**Figure 1**): forward-scattered light (FSC, cell size), side-scattered light (SSC, internal cell structure and granularity), and side fluorescence light intensity (SFL, reflecting DNA/RNA content). Based on the resulting fluorescence and scattered light characteristics, the cells can be separated. For each type of leukocyte (neutrophil, lymphocyte and monocyte), the cell count and CPD were extracted. Neutrophil to lymphocyte ratio (NLR) was calculated.

The CPD included:

- Neutrophil cell complexity (NE-SCC), heterogeneity in cell complexity (NE-WX)
- Fluorescence intensity (NE-SFL), heterogeneity in fluorescence intensity (NE-WY)
- Neutrophil size (NE-FSC) and heterogeneity in size (NE-WZ)
- Lymphocytes cell complexity (LY-X), heterogeneity in cell complexity (LY-WX)
- Fluorescence intensity (LY-Y), heterogeneity in fluorescence intensity (LY-WY)
- Monocytes size (LY-Z) and heterogeneity in size (LY-WZ).
- Monocytes cell complexity (MO-X), heterogeneity in cell complexity (MO-WX)
- Fluorescence intensity (MO-Y), heterogeneity in fluorescence intensity (MO-WY)
- Monocytes size (MO-Z) and heterogeneity in size (MO-WZ).

### Endpoints

The primary endpoint was inflammation-related complication or death occurring during the hospitalisation (6, 7). This endpoint was defined by a composite criteria (8) that included *de novo* supra-ventricular arrythmia, stroke, acute renal failure (defined according to KDIGO guidelines (9)) and/or death.

### Data collection

Baseline demographic data, per-operative and ICU data presented were collected prospectively. The EuroSCORE 2, a validated score for post-operative morbimortality of patients undergoing cardiac surgery was calculated (10). The American Society of Anesthesiologists physical status (ASA score) has been evaluated during the pre-operative consultation (11). Complications were monitored until hospital discharge. All those data were recorded in a data base (Excel, Microsoft). Cell population data are recorded prospectively by the automated hematology analyzer and were extracted retrospectively. The two data bases were merged in order to perform this analysis.

### Statistical analysis

Normality was assessed graphically; quantitative data were presented as means (standard deviation/SD) or medians (interquartile range/IQR) and were compared using the Student t-test and the Kruskall-Wallis nonparametric test. Categorical, ordinal, and binary data were presented as frequencies and percentages and were compared using Chi-squared or Fisher’s exact tests if the conditions of validity were not fulfilled. A Bonferroni correction was applied to compensate for alpha risk inflation with repeated measurements. For longitudinal data, changing patterns of CPD with time were assessed using mixed linear modeling. ROC curve analyses were carried out for cell line parameters of interest. Optimal cut-off values were calculated using Youden’s method. An adjusted logistic regression analysis based on the EuroSCORE 2 was also carried out in order to determine whether those associations were independent of classical risks factors of post-operative morbi-mortality included in this validated score. Last, we developed a multivariable model combining the CPD variable of interest with the EuroSCORE 2 to predict post-operative complications. Data were split into a training set (60%) and a validation set (40%). The model was developed using logistic regression on the training data set. Variable selection was stepwise, AIC based. Then the model accuracy and area under the curve were calculated on the validation set. The Hosmer-Lemeshow Goodness-of-fit Test was also carried out. Missing data were considered to be missing at random and were omitted from the analysis. All analyses were performed using R software.

## Results

### Population and baseline characteristics

**Figure 2:**
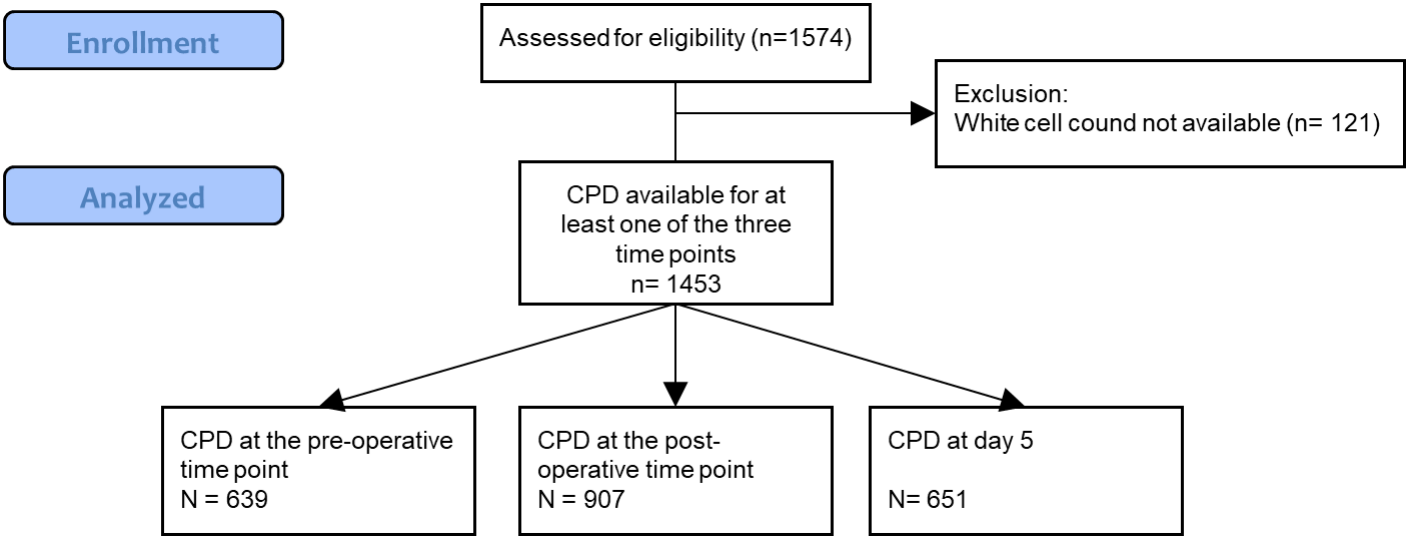
Study flow chart. CPD, Cell Population Data.

After exclusion of 121 patients (**Figure 2**) data from 1453 patients had CPD available for at least one of the time points of interest and were analyzed. Out of those 1453 patients, CPD data were available for 639 patients at the pre-operative time point, 907 patients at the post-operative time points and 651 patients 5 days after surgery. Patients’ characteristics are described in **Table 1**. Median age was 69 [IQR 61;75], most patients were men (76%) with an ASA score of 3 (71.6%) and the median EuroSCORE 2 was 2.23 [IQR 1.19;4.60]. Sixty eight (4.68%) patients had sepsis, mostly due to endocarditis (76%). Among the 636 (43.8%) patients that developed the composite criteria, 342 (23.6%) developed renal failure, 384 (26.4%) *de novo* supra ventricular arrhythmia, 58 (4.0%) stroke and 81 (5.6%) died.

**Table 1:** Characteristics of the patients.

	N= 1453
Baseline	
Age (year)	69.0 [61.0;75.0]
Male (n,%)	1105 (76%)
BMI (Kg/m²)	26.7 [24.2;30.2]
ASA score n,%)	
1	4 (0.28%)
2	173 (11.9%)
3	1039 (71.6%)
4	220 (15.2%)
5	16 (1.10%)
EuroSCORE II	2.23 [1.19;4.60]
Sepsis (n,%)	68 (4.68%)
Per-operative	
Emergent surgery (n,%)	283 (19.5%)
Valvular surgery (n,%)	777 (53.5%)
CABP (n,%)	755 (52.0%)
Combined surgery (n,%)	489 (33.7%)
CPB duration (minutes)	116 [92.0;147]
ICU admission	
SOFA (ICU admission)	5.00 [3.00;6.00]
SAPS-2 (ICU admission)	32.0 [24.0;41.0]
Norepinephrine (ICU admission) (n,%)	1080 (74.3%)
Dobutamine (ICU admission) (n,%)	229 (15.8%)
Outcome	
ICU lengh of stay (days)	2.00 [1.00;4.00]
Hospital lengh of stay (days)	9.00 [7.00;12.0]
In hospital mortality (n,%)	81 (5.57%)
Supra-ventricular arythmia (n,%)	384 (26.4%)
Stroke (n,%)	58 (3.99%)
Acute kidney injury (n,%)	342 (23.5%)

BMI, Body mass index; ASA, American Society of Anesthesiologists; CABP, Coronary artery bypass; CPB, Cardiopulmonary bypass; SOFA, Sequential organ failure assessment; SAPS-2, Simplified acute physiology score-2; ICU, Intensive care unit.

### Neutrophil cell data are modified by cardiac surgery

**Figure 3:**
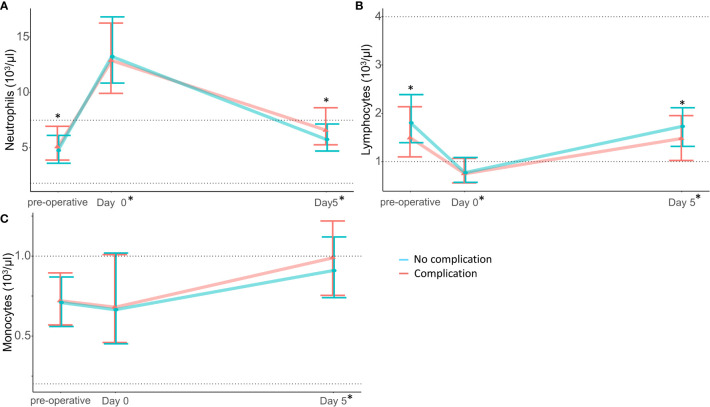
White blood cell count are modified by cardiac surgery and are associated with post-operative complications. **(A)** Neutrophils, **(B)** Lymphocytes, **(C)** Monocytes Dotted line represent normal range for each parameter * above the plot refers between groups significant difference (non-parametric test, p-value were corrected using Bonferroni’s method). * on the x-axis report significant differences from baseline (intragroup comparison with p < 0.05). Red represent patient that developed the composite complication criteria and blue patient who did not.

**Figure 4:**
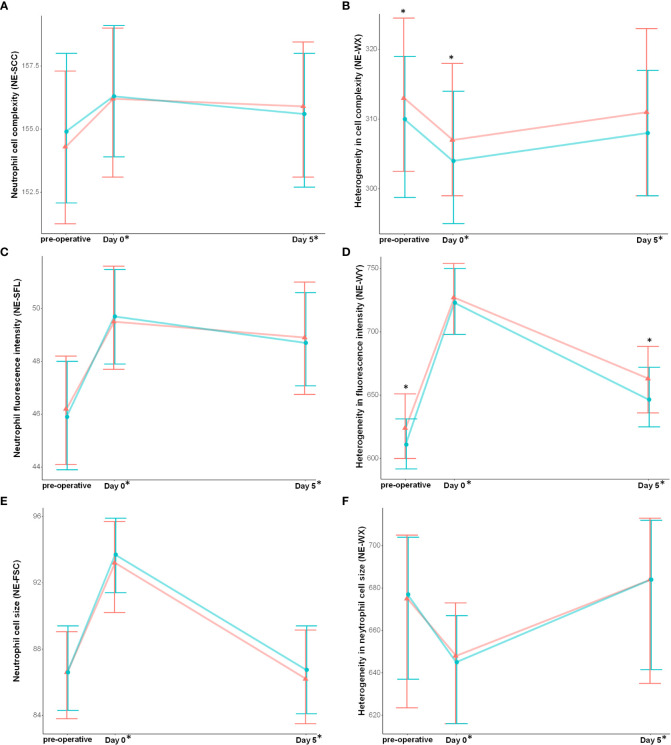
Neutrophil cell populations data are modified with cardiac surgery and are associated with post-operative complications. **(A)** Neutrophil cell complexity, **(B)** Heterogeneity in cell complexity, **(C)** Neutrophil fluorescence intensity, **(D)** Heterogeneity in fluorescence intensity, **(E)** Neutrophil cell size, **(F)** Heterogeneity in neutrophil cell size. Results are presented as median and interquartile range. * above the plot refers between groups significant difference (non-parametric test, p-value were corrected using Bonferroni’s method). * on the x-axis report significant differences from baseline (intragroup comparison with p < 0.05). Red represent patient that developed the composite complication criteria and blue patient who did not.

The neutrophil count (**Figure 3A**), fluorescence granularity (NE-SCC), intensity (NE-SFL) and size (NE-FSC) increased with surgery (**Figures 4A, C, E** respectively). Heterogeneity of neutrophils decreased in terms of fluorescence granularity (NE-WX) and size (NE-WZ) but increased in terms of intensity (NE-WY) (**Figures 4B, D, F** respectively).

### Neutrophil cell data are associated with post-operative complications

Patients who developed the composite complication score had higher pre-operative neutrophil counts (5.08 [3.89;6.95] vs 4.76 [3.60;6.13] p = 0.02) and more heterogeneous neutrophils in terms of fluorescence granularity (NE-WX, p< 0.01) and intensity (NE-WY, p< 0.01) (**Figures 4B, D** and **Table 2**). Immature granulocytes and the neutrophil to lymphocyte ratio (NLR) increased with cardiac surgery, and pre-operative NLR was associated with occurrence of post-operative complications (p<0.01 for both) (**Supplementary Table 1**). Using multivariate analysis, NE-WX and NE-WY were associated with post-operative complications independently of the EuroSCORE 2 (adjusted odd ratio of 1.01 [1.00;1.02] and 1.01 [1.00;1.01] respectively) (**Table 2**).

**Table 2:** Area under the curve, threshold and odd ratio adjusted for EuroSCORE 2.

Variable	AUC (95% CI)	Optimal threshold	Accuracy	OR adjusted for the EuroSCORE 2
Neutrophil count	0.56 [0.51;0.60]	6.65	0.52 [0.50;0.54]	1.02 [0.97;1.08]
NE-WX	0.57 [0.52;0.62]	320	0.52 [0.50;0.54]	1.01 [1.00;1.02]*
NE-WY	0.61 [0.56;0.65]	636	0.53 [0.51;0.55]	1.01 [1.00;1.01]*
Lymphocyte count	0.61 [0.57;0.66]	1.52	0.53 [0.51;0.56]	0.93 [0.80;1.08]
LY-X	0.57 [0.53;0.62]	80.2	0.52 [0.50;0.54]	1.08 [1.02;1.15]*
Immature granulocyte	0.57 [0.53;0.61]	0.06	0.52 [0.50;0.54]	2.27 [0.67;15.24]
Neutrophyl to Lymphocyte ratio	0.61 [0.57;0.66]	3.5	0.53 [0.51;0.55]	1.05 [1.01;1.10]*

OR, Odd ratio. For adjusted OR, each parameter was individually adjust on the EuroSCORE 2. Accuracy is given for the optimal threshold. *p <0.05.

### Lymphocyte cell data are modified by cardiac surgery

**Figure 5:**
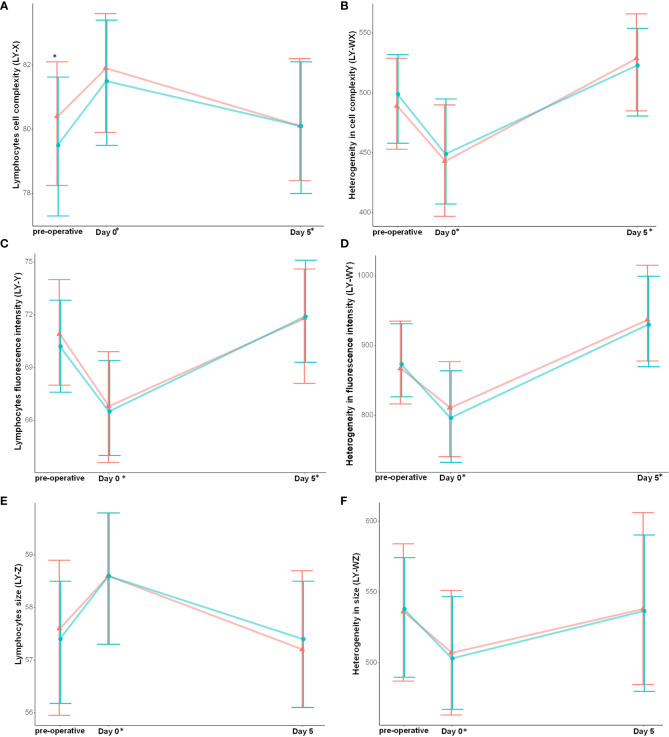
Lymphocyte cell populations data are modified with cardiac surgery and are associated with post-operative complications. **(A)** Lymphocytes cell complexity, **(B)** Heterogeneity in cell complexity, **(C)** Lymphocytes fluorescence intensity, **(D)** Heterogeneity in fluorescence intensity, **(E)** Lymphocytes cell size, **(F)** Heterogeneity in lymphocytes cell size. Results are presented as median and interquartile range. *: between groups significant difference (non-parametric test, p-value were corrected using Bonferroni’s method). * on the x-axis report significant differences from baseline (intragroup comparison with p < 0.05). Red represent patient that developed the composite complication criteria and blue patient who did not.

The lymphocyte count decreased with surgery (**Figure 3B**). While fluorescence granularity (LY-X) and size (LY-Z) increased, lymphocyte fluorescence intensity decreased (LY-Y) (**Figures 5A, C, E**). Lymphocytes were less heterogeneous in terms of their granularity, intensity and size after surgery (i.e., lower LY-WX, LY-WY and LY-WZ respectively).

### Lymphocyte cell data are associated with post-operative complications

Patients who developed post-operative complications had lower pre-operative lymphocyte counts (1.49 [1.10;1.14] vs 1.81 [1.39;2.39], p<0.01) and fluorescence granularity (LY-X, p<0.01)) (**Figure 5**). Using multivariate analysis, LY-X was associated with the occurrence of post-operative complications, independently of the EuroSCORE 2 (adjusted odd ratio of 1.08 [1.02;1.15]) (**Table 2**).

### Monocyte cell data are not associated with post-operative complications

The monocyte count was unchanged after cardiac surgery (**Figure 3C**). There was no difference between patients developing complications and others in terms of pre-operative monocyte cell parameters (**Supplementary Table 2**).

### Multivariate model for post-operative complications

After stepwise variable selection (training data set), EuroSCORE 2, NE-WY and LY-X were kept in the model (Supp file 3). The accuracy of this model (testing data set) was of 0.65 [0.59;0.71] and the Area under the curve (AUC) of 0.63 [0.58;0.68]. The p-value of the Hosmer-Lemeshow Goodness-of-fit Test was of 0.33. For the EuroSCORE 2 alone the accuracy was of 0.63 [0.56:0.69] and the AUC of 0.60 [0.55;0.65].

## Discussion

Our main finding is that CPD were highly modified by the systemic aggression caused by cardiac surgery and cardiopulmonary bypass. In addition, we were able to identify pre-operative differences in CPD associated with post-operative complications. In particular: a high pre-operative neutrophil count, a low per-operative lymphocyte count, and a high immature granulocyte count were associated with the occurrence of post-operative complications. Neutrophils were also more heterogenous in granularity and intensity and lymphocytes had lower granularity in patients who developed complications. CPD are highly modified in the context of sterile inflammation, further supporting the use of CPD as a marker of immune response during critical illness.

In patients undergoing cardiac surgery, the pre-operative inflammatory status is a major concern and influences clinical outcome (12). Among the traditional blood cell count data reported, high neutrophil and low lymphocyte counts have been related to adverse outcomes following coronary graft surgery (13, 14). In addition to a pro-inflammatory state, a low lymphocyte count has also been suggested to reflect poorer general health (15) which might also link lymphopenia to worse outcome. In line with this, by combining these two markers, the neutrophil to lymphocyte ratio (NLR) has been reported as a predictor of morbidity and mortality in patients undergoing cardiac surgery (15, 16). Our results are consistent with those findings and further support the importance of the pre-operative inflammatory status.

In addition to the traditional quantitative leukocyte count, the automated blood analyzer provides data that give qualitative information about leukocytes (cell fluorescence intensity, granularity, size and the heterogeneity of these three parameters among each cell line (17)) that could be used to greater advantage. Neutrophil fluorescence intensity (NE-SFL) and the heterogeneity in fluorescence (NE-WY) have been demonstrated to discriminate sepsis with a good accuracy, leading to hypothesize that these parameters indicate neutrophil activation or immaturity (18). NE-SFL is related to the degree of chromatin, DNA and RNA condensation and thus is likely to indicate neutrophil cell activation, i.e., in transcriptional activity. The increase in neutrophil size observed in our cohort could indicate increased neutrophil heterogeneity that could be linked to bone marrow release of immature granulocytes. The increase of NE-WY, reflecting the fluorescent light distribution width of the neutrophil area may also indicate neutrophil immaturity. Using multivariate analysis, the heterogeneity of neutrophils (NE-WX, NE-WY) and Lymphocytes cell complexity (LY-X) were associated with the occurrence of complications after adjustment on the EuroSCORE 2, suggesting that these parameters could bring additional information to traditional complication scores and be useful in early detection of complications.

Cardiac surgery with cardiopulmonary bypass triggers inflammation by multiple mechanisms (19). As in sepsis, during cardiac surgery, inflammation drives adverse clinical outcome (20, 21). However, this inflammation is unrelated to pathogen aggression and involves different pathways (22). Leucocytes counts modifications induced by CPB (increased neutrophil, decrease lymphocyte and similar monocyte) reported in our study are in line with previous results (23). Nevertheless, our study provides new additional information on blood cell properties and activation induced by cardiac surgery with cardiopulmonary bypass. In our data, whereas baseline NE-SFL and NE-WY were in the same range of values as healthy controls, the post-operative values increased but were not as elevated as those reported in patients with sepsis (4, 18). The heterogeneity in neutrophil CPD probably reflects different states of leukocyte activation, further underlying the separate immune pathways of sterile and septic inflammation. Thus, CPD may represent a new tool for a better understanding of immune cell activation during critical illness. As blood counts are widely available, CPD could provide interesting additional information for the monitoring of immunity.

This study has some limitations. Firstly, this is an observational study and only association could be inferred. Secondly, as leukocyte count is not routinely performed daily, there was a large amount of missing data which might introduce a selection bias. Indeed, patients with a pre-operative blood cell count seemed to be more severe at baseline than patients with missing data (**Supplementary Table 3**). Blood transfusion might induce significant modification of CPD kinetics. Complications were only monitored until hospital discharge, thus only “early” complications are reported in this analysis. Lastly, biomarkers of inflammation were not measured in those post-operative patients and could have provide interesting additional information.

In conclusion, CPD were highly modified by cardiac surgery with cardiopulmonary bypass and some of those modifications were associated with post-operative complications, suggesting that CPD reflect the activation of leukocytes in sterile inflammation. Before surgery: NE-WX, NE-WY and LY-X were associated with post-operative complication independently of the EuroSCORE 2. Those parameters measured with traditional blood cell counts might represent a readily available and low cost tool for the monitoring of immune function during critical illness. Our findings requires validation on a larger external cohort.

## Data availability statement

The raw data supporting the conclusions of this article will be made available by the authors, without undue reservation.

## Ethics statement

The studies involving human participants were reviewed and approved by “Comité d’Ethique pour la Recherche en Anesthésie-Réanimation”. Written informed consent for participation was not required for this study in accordance with the national legislation and the institutional requirements.

## Author contributions

MN, MB, AG, DM, LS, P-GG contributed to the study design. MN, BD, AG, MB, P-AB, CF collected the data. MN, BB P-GG, LS analysed the data. MN, LS drafted the manuscript. MN, LS, AG, P-AB, DM, CT, MB, JG, CF, BD, BB, P-GG reviewed and edited the manuscript. All authors contributed to the article and approved the submitted version.
