# Supplementary material for: Leukocyte cell population data in patients with cardiac surgery and cardiopulmonary bypass: A potential readily available tool to monitor immunity
Source: Front Immunol. 2023 Jan 19;13:1101937. doi: 10.3389/fimmu.2022.1101937 (PMC9892932; doi:10.3389/fimmu.2022.1101937)
Supplement: Supplementary file 1 [file DataSheet_1.docx]

|  | No complication  817 | Complication  636 | p |
| --- | --- | --- | --- |
| **Immature Granulocyte count: 10^3^/µl** | | | |
| Pre-operative | 0.03 [0.02;0.05] | 0.03 [0.02;0.06] | <0.01 |
| Post-operative* | 0.11 [0.08;0.20] | 0.12 [0.07;0.19] | 1.00 |
| Day 5* | 0.08 [0.05;0.17] | 0.11 [0.06;0.23] | <0.01 |
| **Neutrophil to Lymphocyte ratio** | | | |
| Pre-operative | 2.54 [1.75 ;3.40] | 3.28 [2.12 ;5.23] | <0.01 |
| Post-operative* | 18.1 [11.6 ;23.9] | 16.6 [11.3 ;23.8] | 0.42 |
| Day 5 * | 3.47 [2.64 ;4.37] | 4.54 [3.24 ;6.66] | <0.01 |

**Supp file 1.** Immature granulocyte count

p-refers to between group comparison. * refers to intra-group comparison (difference from baseline).


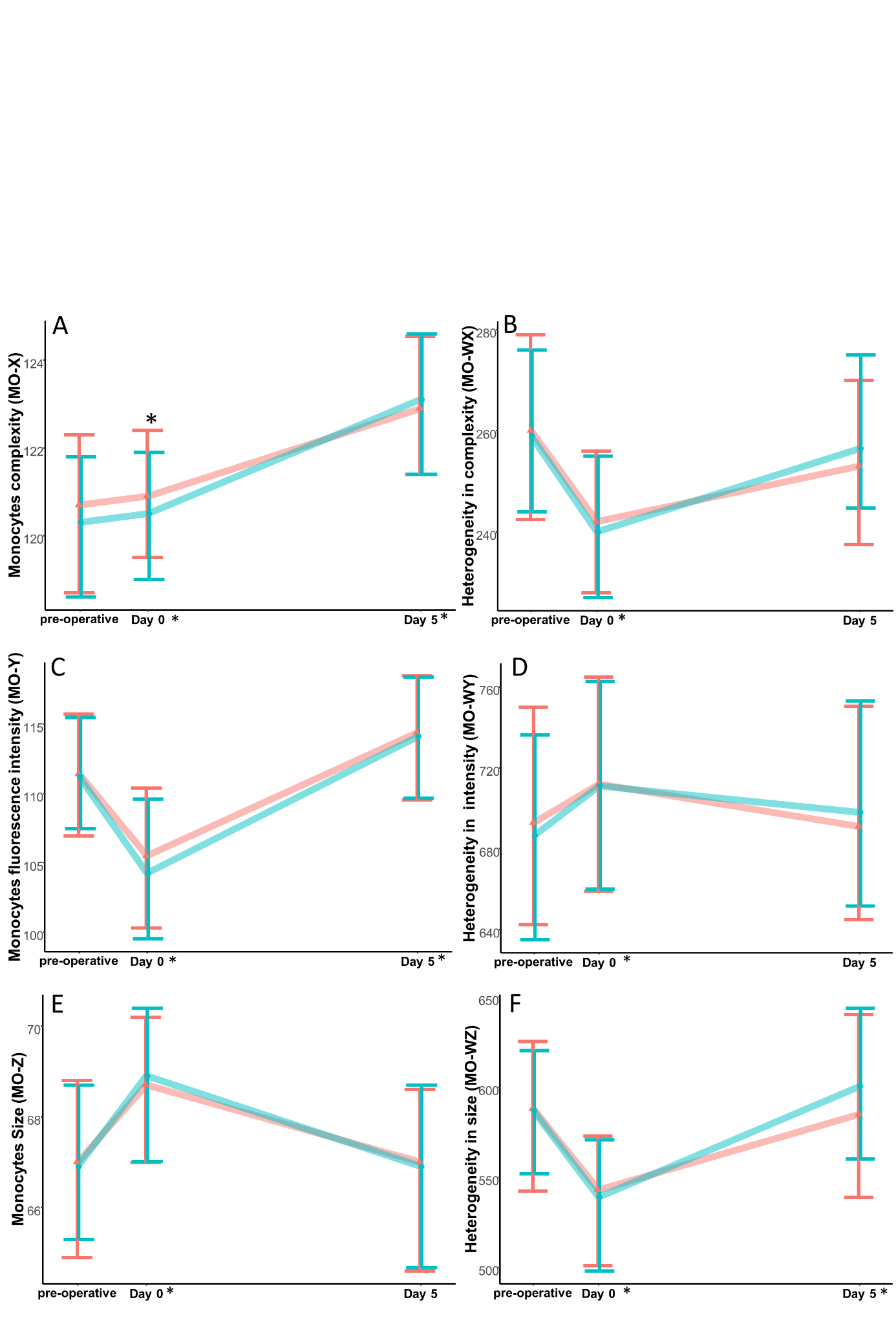


**Supp file 2.** Monocyte cell line characteristics.

* above the plot refers between groups significant difference (non-parametric test, p-value were corrected using Bonferroni’s method). * on the x-axis report significant differences from baseline (intragroup comparison with p < 0.05). Red represent patient that developed the composite complication criteria and blue patient who did not.

| **Variable** | **Adjusted odd ratio** | **p** | |
| --- | --- | --- | --- |
| EuroSCORE 2 | 1.08 [1.05;1.12] | | <0.01 |
| LY-X | 1.08 [1.01;1.16] | | 0.04 |
| NE-WY | 1.00 [1.00;1.01] | | 0.14 |

**Supp file 3.** Multivariate modeling to prevent the composite complication score.

|  | Pre-operative sample  N= 639 | No-preoperative sample  N= 814 |
| --- | --- | --- |
| Age (year) | 69.0 [62.0;75.0] | 69.0 [61.0;74.0] |
| Male (n,%) | 496 (77.6%) | 609 (74.8%) |
| BMI (Kg/m²) | 26.8 [24.1;30.4] | 26.6 [24.2;30.1] |
| ASA score |  |  |
| 1 | 0 (0.00%) | 4 (0.49%) |
| 2 | 50 (7.82%) | 123 (15.1%) |
| 3 | 430 (67.3%) | 609 (74.9%) |
| 4 | 148 (23.2%) | 72 (8.86%) |
| 5 | 11 (1.72%) | 5 (0.62%) |
| Euroscore II | 2.94 [1.52;6.85] | 1.83 [1.05;3.46] |
| Sepsis | 61 (9.55%) | 7 (0.86%) |

**Supp file 4.** Baseline characteristics depending on the presence of a pre-operative blood cell count

BMI: Body mass index, ASA: American Society of Anesthesiologists
